# Supplementary material for: Accurate prediction of ecDNA in interphase cancer cells using deep neural networks
Source: Commun Biol. 2026 Apr 11;9:805. doi: 10.1038/s42003-026-09982-4 (PMC13265960; doi:10.1038/s42003-026-09982-4)
Supplement: Supplementary file 1 — Supplementary Information [file 42003_2026_9982_MOESM1_ESM.pdf]

Supplementary Figure Captions

Supplementary Figure 1: Examples of images from each acquisition protocol. (a) Image from the cultured cell line GBM39EC (*EGFR* red, Centromere 7 green). (b) Image from the tissue model cell line DLD1 (*MYC* red, Centromere 8 green). (c) Image from a neuroblastoma patient tissue sample (*MCYN* green, Centromere 2 red).

Supplementary Figure 2: EcSeg-c Architecture Diagram. EcSeg-c architecture based on DenseNet-121.

Supplementary Figure 3: EcSeg-i's filters and feature maps. Top left shows a sample image (*MYCN* red and Centromere 2 green) that is passed to ecSeg-i. Top right shows filters from the first convolutional layer. Bottom row shows the feature maps produced by convolving the filters over the sample image. The ecSeg-i prediction for this nucleus is EC-amp.

Supplementary Figure 4: InterSeg evaluation on images with shrinking distortion. The top row shows the original HSR-amp image, the shrunken image, and the interSeg predictions for both. The bottom row shows the original EC-amp image, the shrunken image, and the bootstrapped interSeg predictions for both.

Supplementary Figure 5: InterSeg evaluation on images with enlarging distortion. The top row shows the original HSR-amp image, the enlarged image, and the interSeg predictions for both. The bottom row shows the original EC-amp image, the enlarged image, and the bootstrapped interSeg predictions for both.

Supplementary Figure 6: InterSeg evaluation on images with rotation distortion. The top row shows the original HSR-amp image, the rotated image, and the interSeg predictions for both. The bottom row shows the original EC-amp image, the rotated image, and the bootstrapped interSeg predictions for both.

Supplementary Figure 7: InterSeg evaluation on HSR-amp images with contrast distortion. The top row shows the original HSR-amp image, a low contrast version, and a high contrast version. The middle row displays the pixel distribution of the blue and red color channels to quantify the contrast. The bottom row shows the bootstrapped interSeg predictions, with 'n' representing the number of cells analyzed.

Supplementary Figure 8: InterSeg evaluation on EC-amp images with contrast distortion. The top row shows the original EC-amp image, a low contrast version, and a high contrast version. The middle row displays the pixel distribution of the blue and red color channels to quantify the contrast. The bottom row shows the bootstrapped interSeg predictions, with 'n' representing the number of cells analyzed.

Supplementary Figure 9: Stat-FISH pipeline. Visualization of stat-FISH pipeline and sample analysis.

Supplementary Figure 10: Comparison of NuSeT Auto Segmentation with Manual Segmentation. (a) A neuroblastoma patient tissue image from sample CC, annotated as 'amplification' by pathologists. The *MYCN* oncogene and Centromere 2 FISH probes are shown in green and red, respectively. Segmentation boundaries are marked in purple. The left image displays the manual segmentation boundaries, and the right image shows the automated NuSeT segmentation boundaries. (b) A neuroblastoma patient tissue image from sample DB, annotated as 'no amplification' by pathologists. The *MYCN* oncogene and Centromere 2 FISH probes are shown in green and red, respectively. Segmentation boundaries are marked in purple. The left image displays the manual segmentation boundaries, and the right image shows the automated NuSeT segmentation boundaries. (c) The raw (non-bootstrapped) interSeg predictions for the 'amplification' annotated samples CC and KM. (d) The raw (non-bootstrapped) interSeg predictions for the 'no amplification' annotated samples DB and DL.

Supplementary Figure 11: InterSeg performance without bootstrapping. (a) F1-score on the cultured and tissue model test set, where n is the number of cells in each class. (b) Raw (non-bootstrapped) distribution of amplification mechanism of no-amp cell lines. (c) Raw distribution of amplification mechanism of EC-amp cell lines. (d) Raw distribution of amplification mechanism of HSR-amp cell lines.

Supplementary Figure 12: InterSeg prediction of ecDNA in SF268 interphase cells. (a) A metaphase spread of SF268 containing interphase nuclei. The *MMP8* oncogene is visualized by the red FISH probe, while the pan-centromeric probe is shown by the green FISH probe. (b) The left image displays a zoomed-in view of a selected interphase nucleus, with the corresponding interSeg prediction annotated. The right image displays the segmented nucleus, showing peaks of the oncogene and centromere signals as identified by stat-FISH. For this selected interphase nucleus, Stat-FISH predicted 7 *MMP8* foci. (c) A zoomed-in region of the metaphase spread, displaying 2 intrachromosomal *MMP8* amplification signals.

Supplementary Figure 13: InterSeg prediction of HSR-amp in SF268 interphase cells. (a) A second metaphase spread of SF268 containing interphase nuclei. The *MMP8* oncogene is visualized by the red FISH probe, while the pan-centromeric probe is shown by the green FISH probe. (b) The left image displays a zoomed-in view of a selected interphase nucleus, with the corresponding interSeg prediction annotated. The right image displays the segmented nucleus, showing peaks of the oncogene and centromere signals as identified by stat-FISH. For this selected interphase nucleus, Stat-FISH predicted 9 *MMP8* foci. (c) A zoomed-in region of the metaphase spread, displaying 2 intrachromosomal *MMP8* amplification signals.

Supplementary Figure 14: InterSeg prediction of ecDNA in SN12C interphase cells. (a) A metaphase spread of SN12C containing interphase nuclei. The *TNFRSF13B* oncogene is visualized by the red FISH probe; the green FISH probe is pan-centromeric. (b) The left image displays a zoomed-in view of a selected interphase nucleus, with the corresponding interSeg prediction annotated. The right image displays the segmented nucleus, showing peaks of the oncogene and centromere signals as identified by stat-FISH. For this selected interphase

nucleus, Stat-FISH predicted 4 *TNFRSF13B* foci. (c) A zoomed-in region of the metaphase spread, displaying 4 intrachromosomal *TNFRSF13B* amplification signals.

Supplementary Figure 15: Visualization of heterogeneous mix of COLO320DM and COLO320HSR. The top row of images depicts 3 nuclei in the hybrid COLO320DM and COLO320HSR plate with the mCherry RFP probe in red and the DNA-FISH probe for *MYC* in green. The bottom row displays only the mCherry RFP probe in grayscale, with the color bar displaying observed pixel intensity. Based on the distribution of the green FISH-probe for *MYC*, the nucleus in the left column appears to be HSR-amplified, while the middle and right column nuclei appear to be EC-amplified. While the middle column nucleus displays a high mCherry signal, the right column nucleus displays a low mCherry signal.

Supplementary Figure 16: InterSeg prediction accuracy in heterogeneous mix of COLO320DM and COLO320HSR. The left and right boxplots display the precision vs recall curves for mCherry tagged nuclei (COLO320DM) and not mCherry tagged nuclei (COLO320HSR), respectively. Each point represents a threshold for the maximum mCherry brightness per nuclei. Nuclei below the threshold were labeled as not mCherry tagged (HSR-amp) and the remaining nuclei were labeled as mCherry tagged (EC-amp). For each choice of threshold, the mCherry tagging status was used as a gold-standard to measure the accuracy of interSeg prediction.

Supplementary Figure 17: InterSeg prediction on neuroblastoma hold-out set images without bootstrapping. Non-bootstrapped distribution of interSeg amplification mechanism across the 67 NB hold-out test set images, stratified by the pathologist annotated ‘amplification’ and 'No amplification' labels. Each column corresponds to a single patient, and the bar height corresponds to the proportion of cells labeled for each amplification class by interSeg.

Supplementary Figure 1

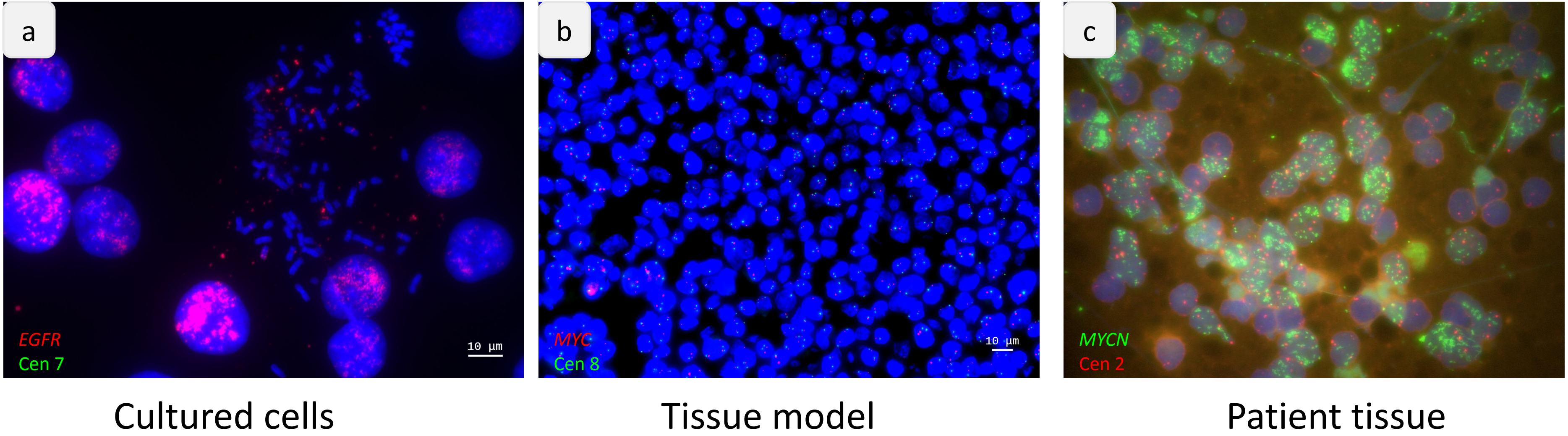

Supplementary Figure 2

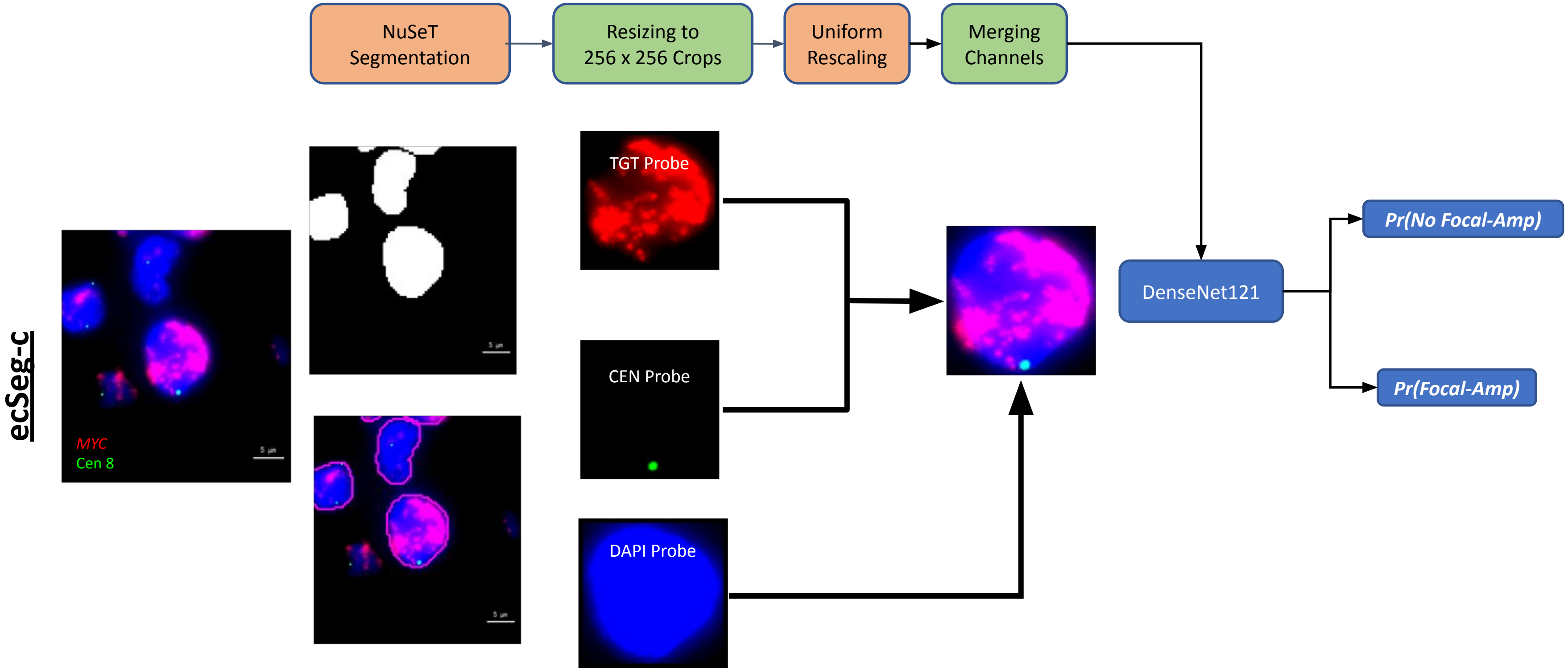

Supplementary Figure 3

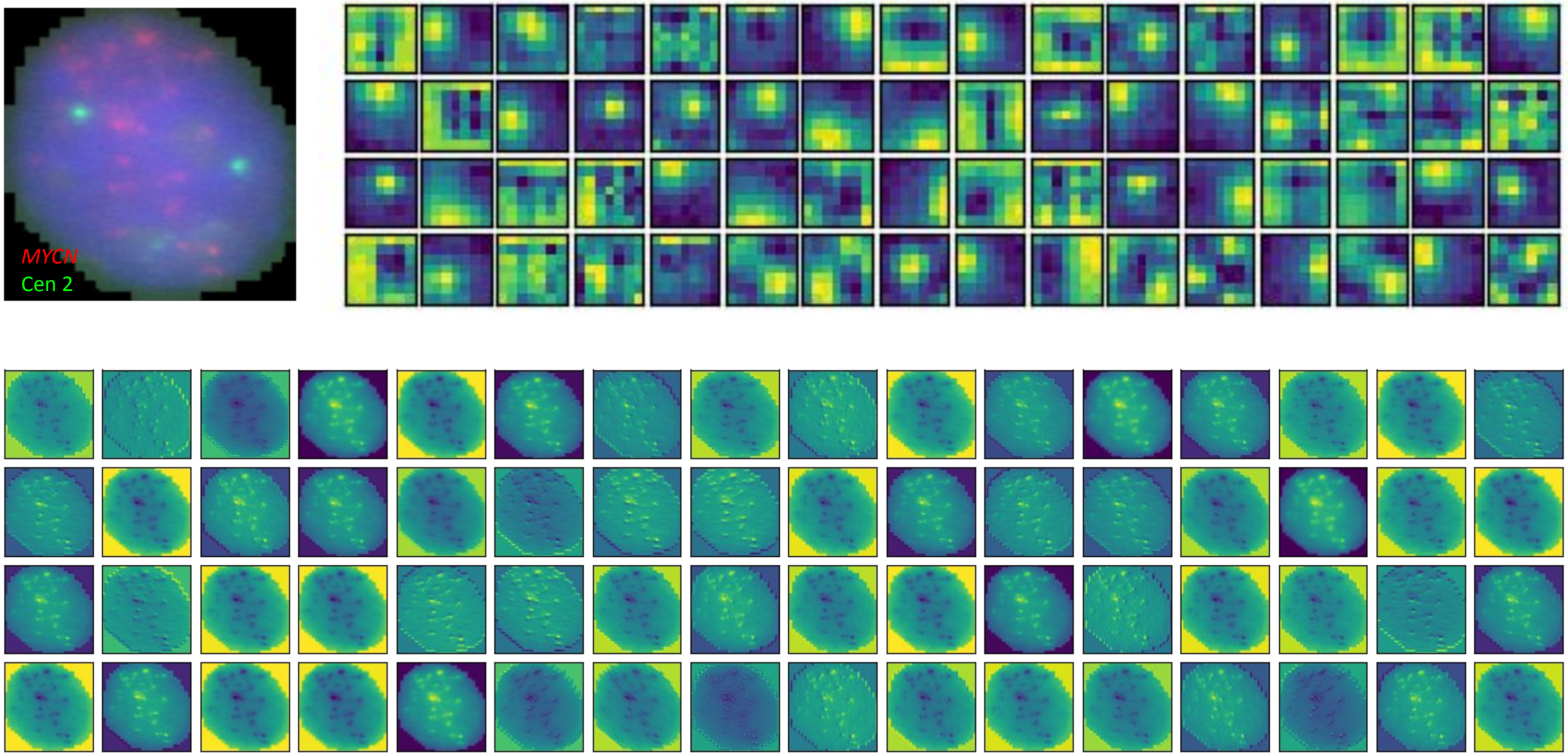

Supplementary Figure 4

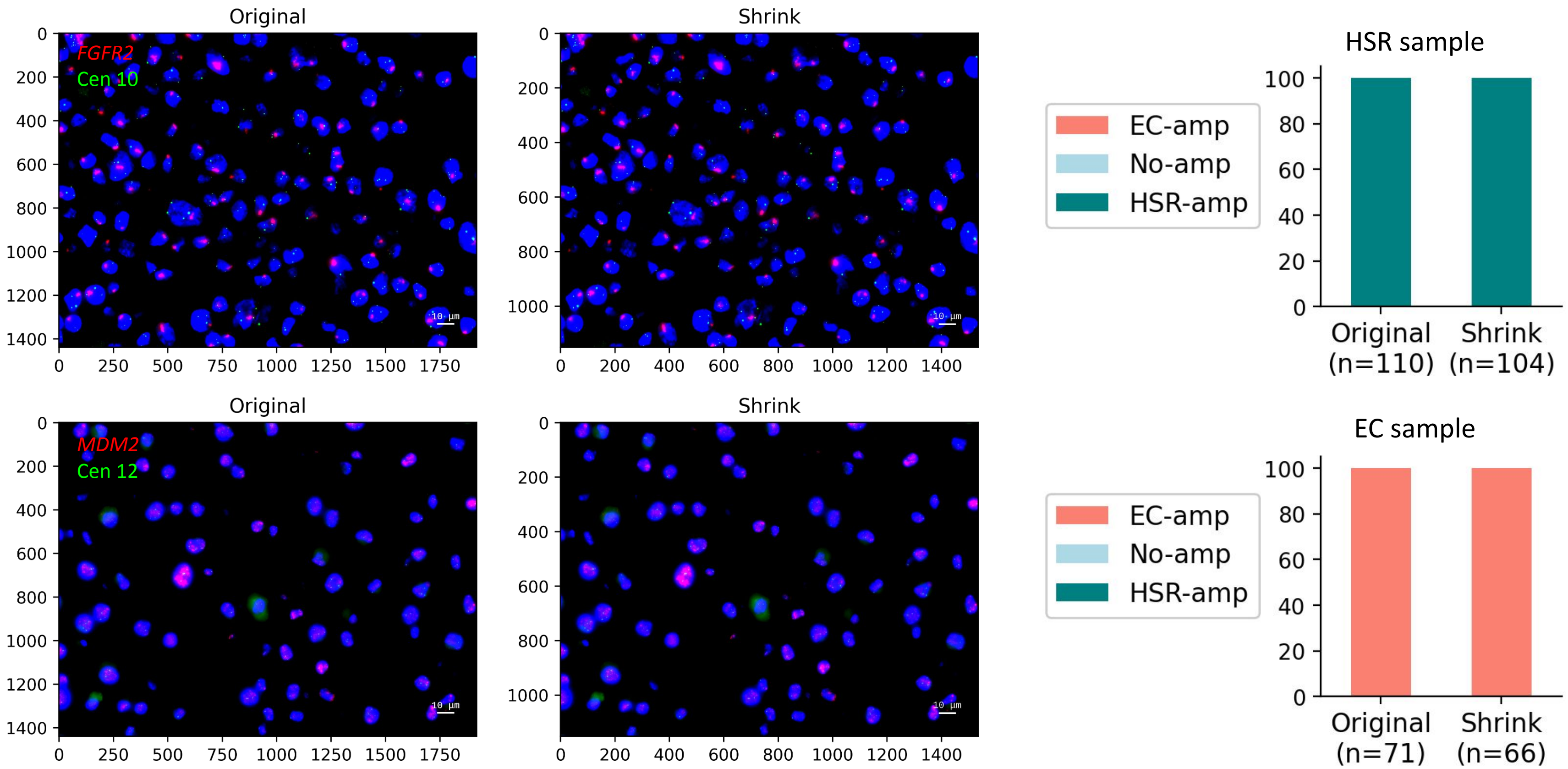

Supplementary Figure 5

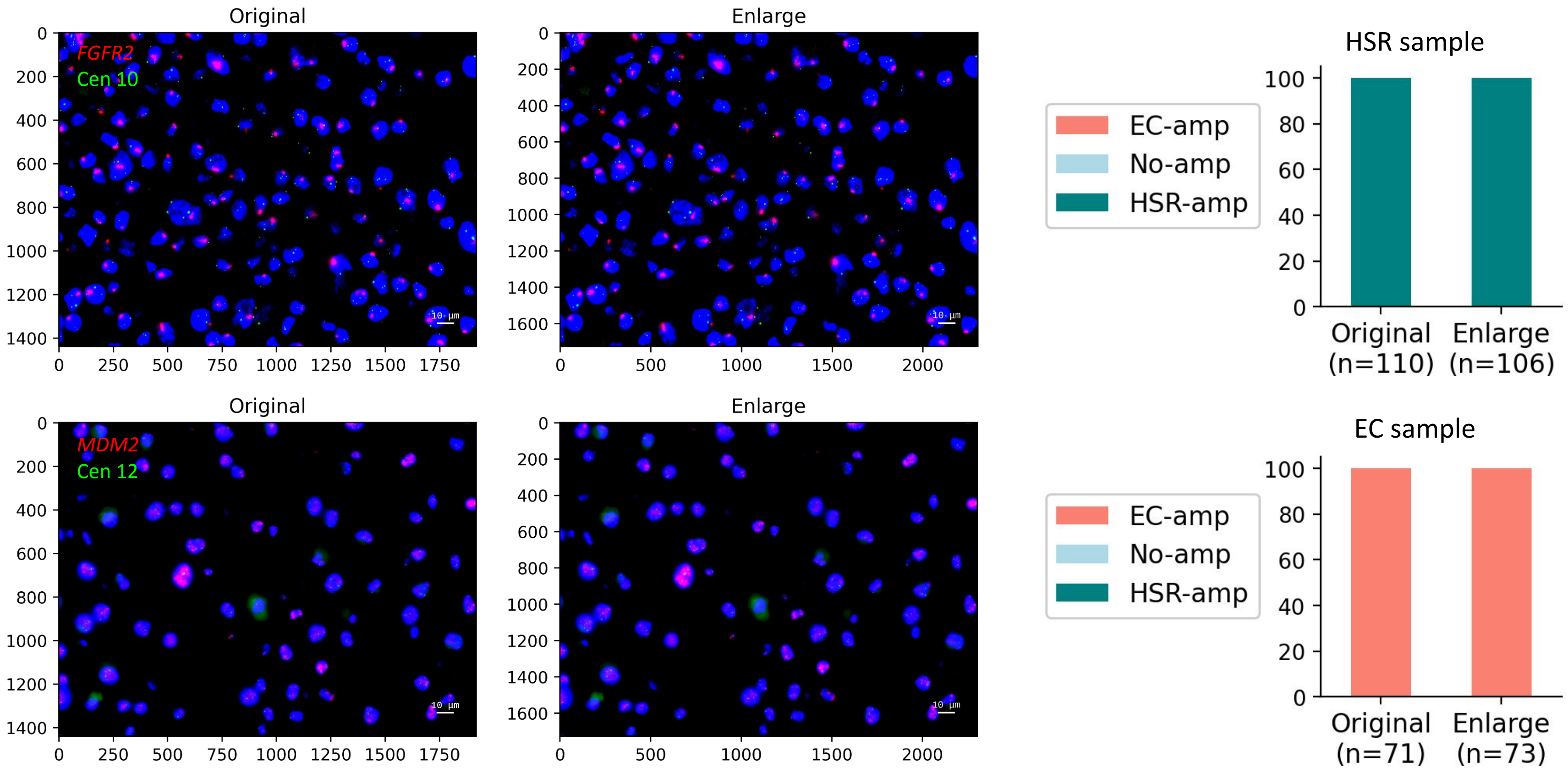

Supplementary Figure 6

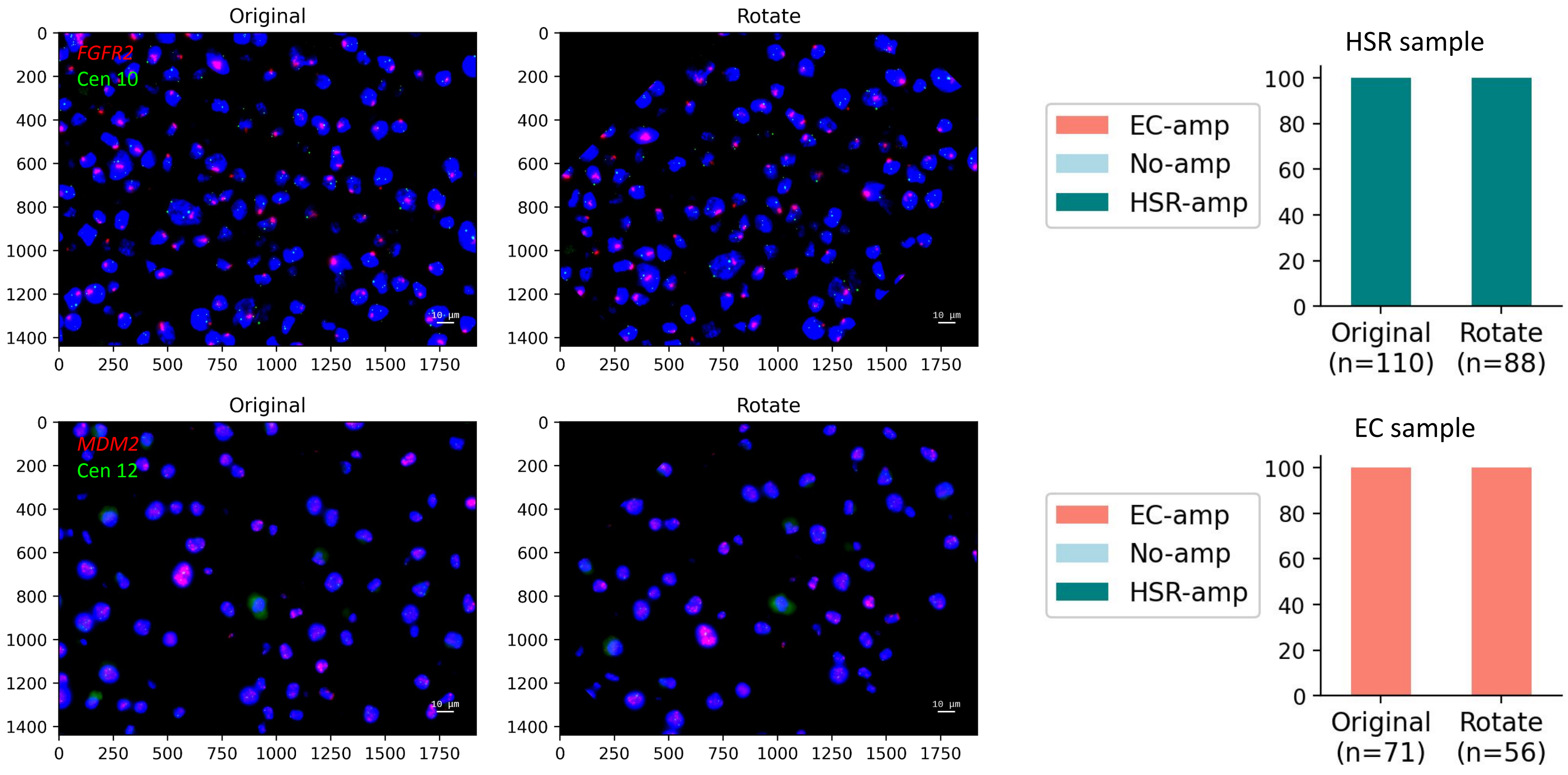

Supplementary Figure 7

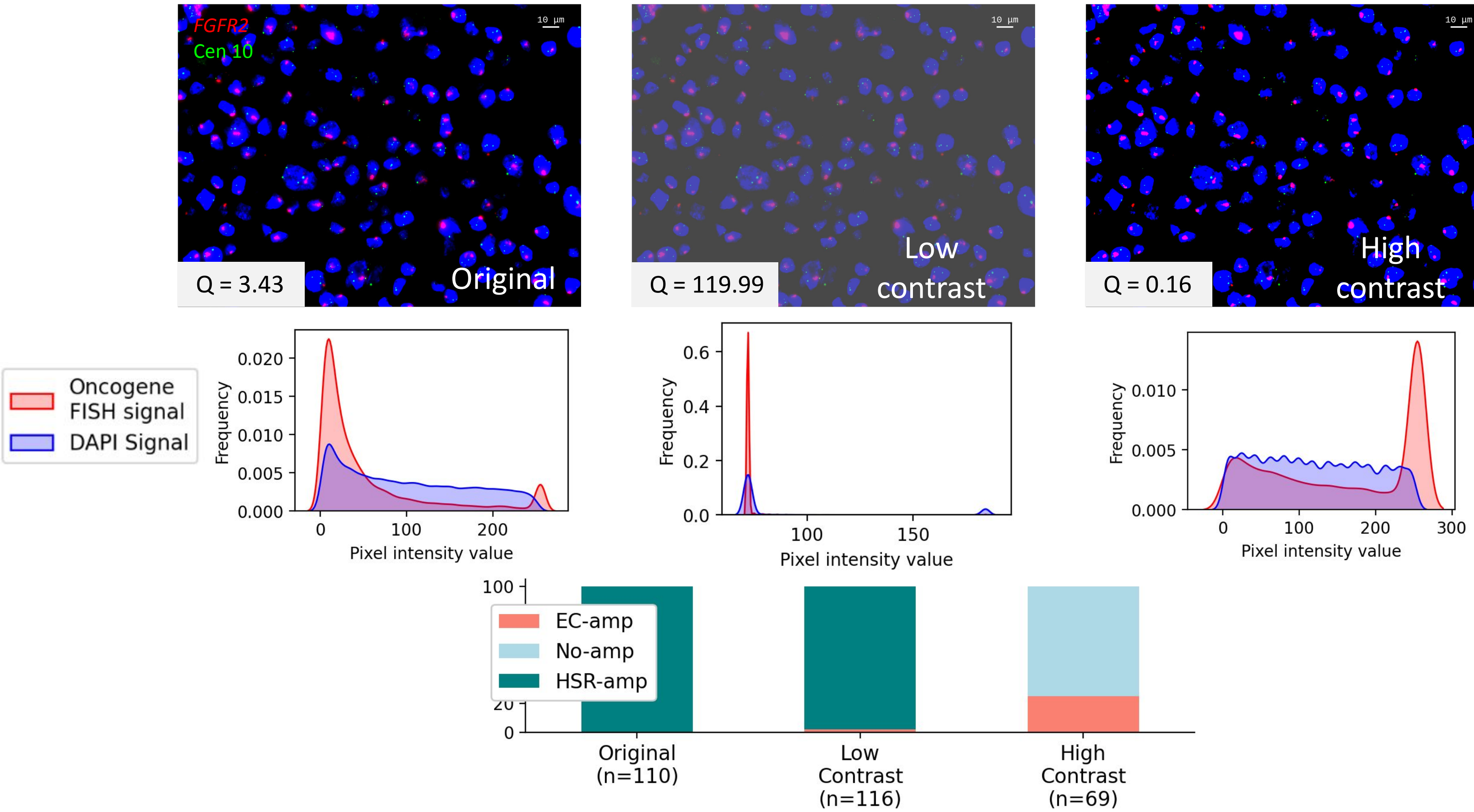

Supplementary Figure 8

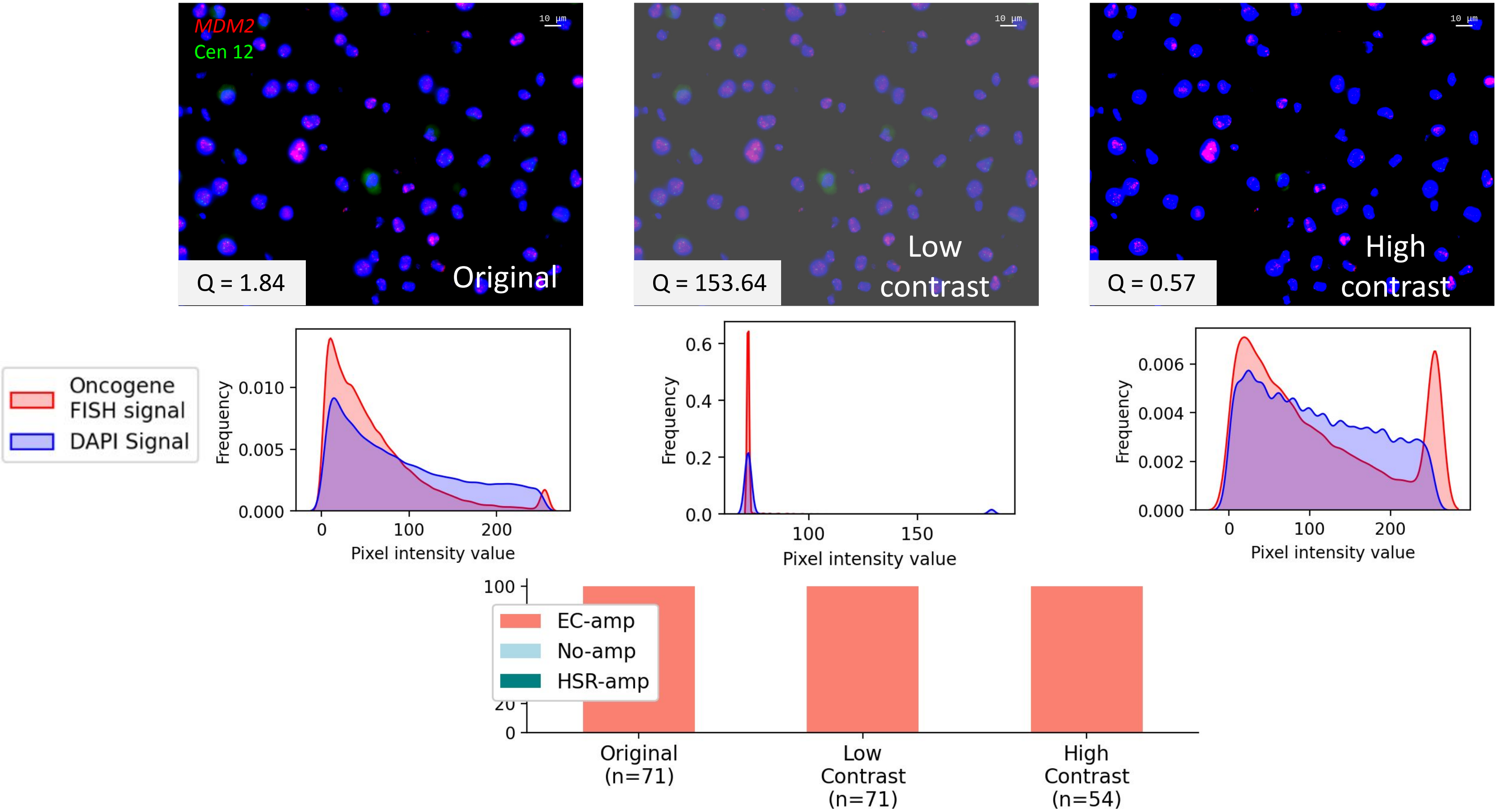

Supplementary Figure 9

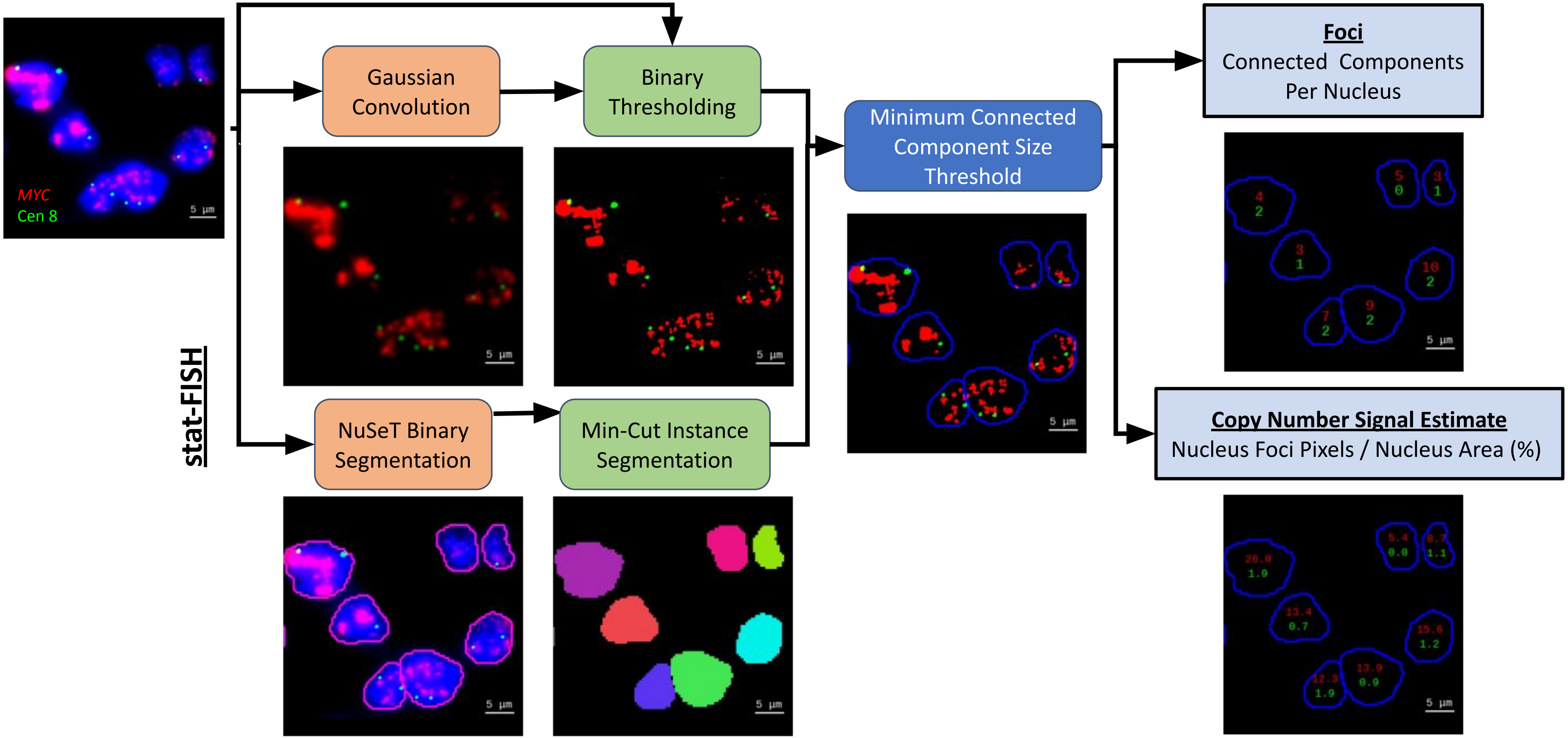

Supplementary Figure 10

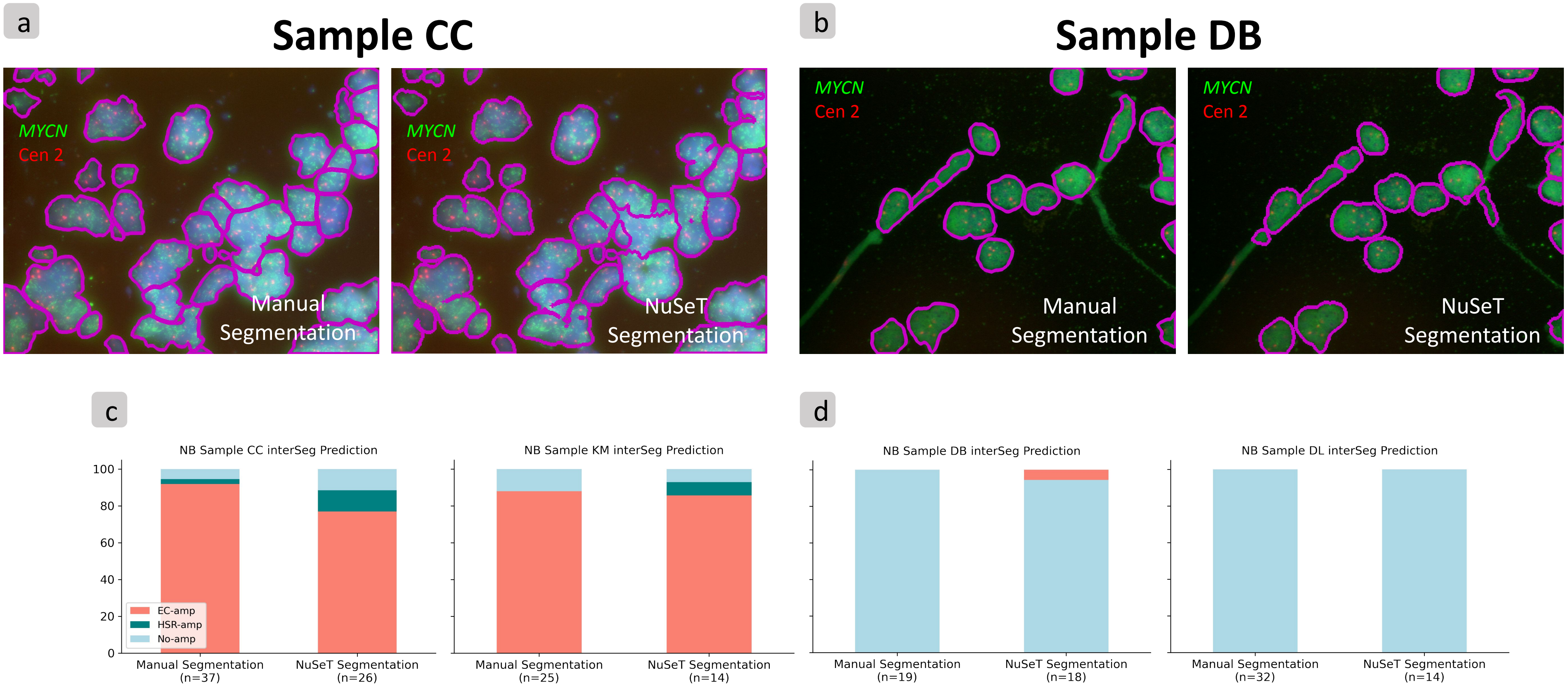

Supplementary Figure 11

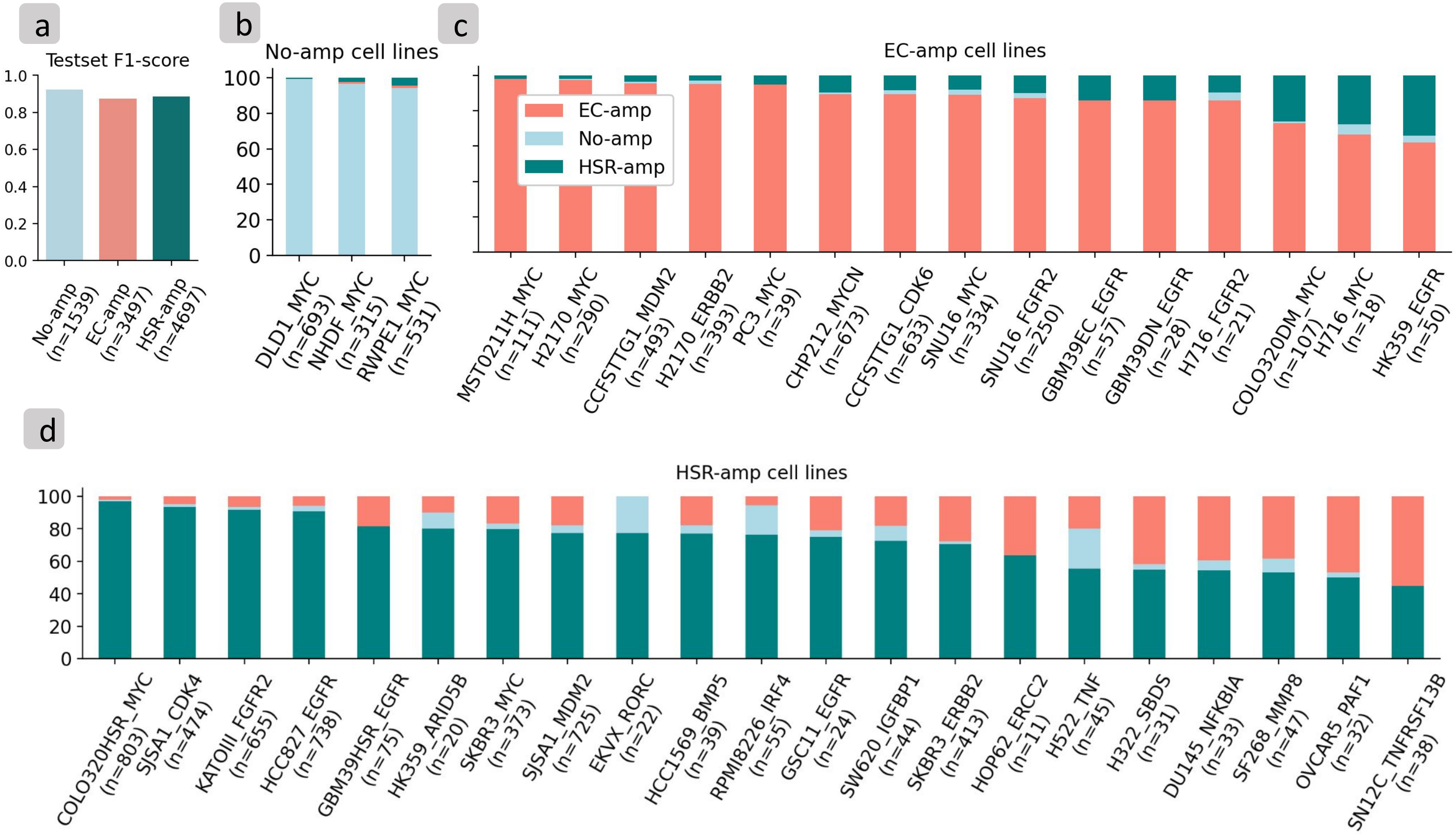

Supplementary Figure 12

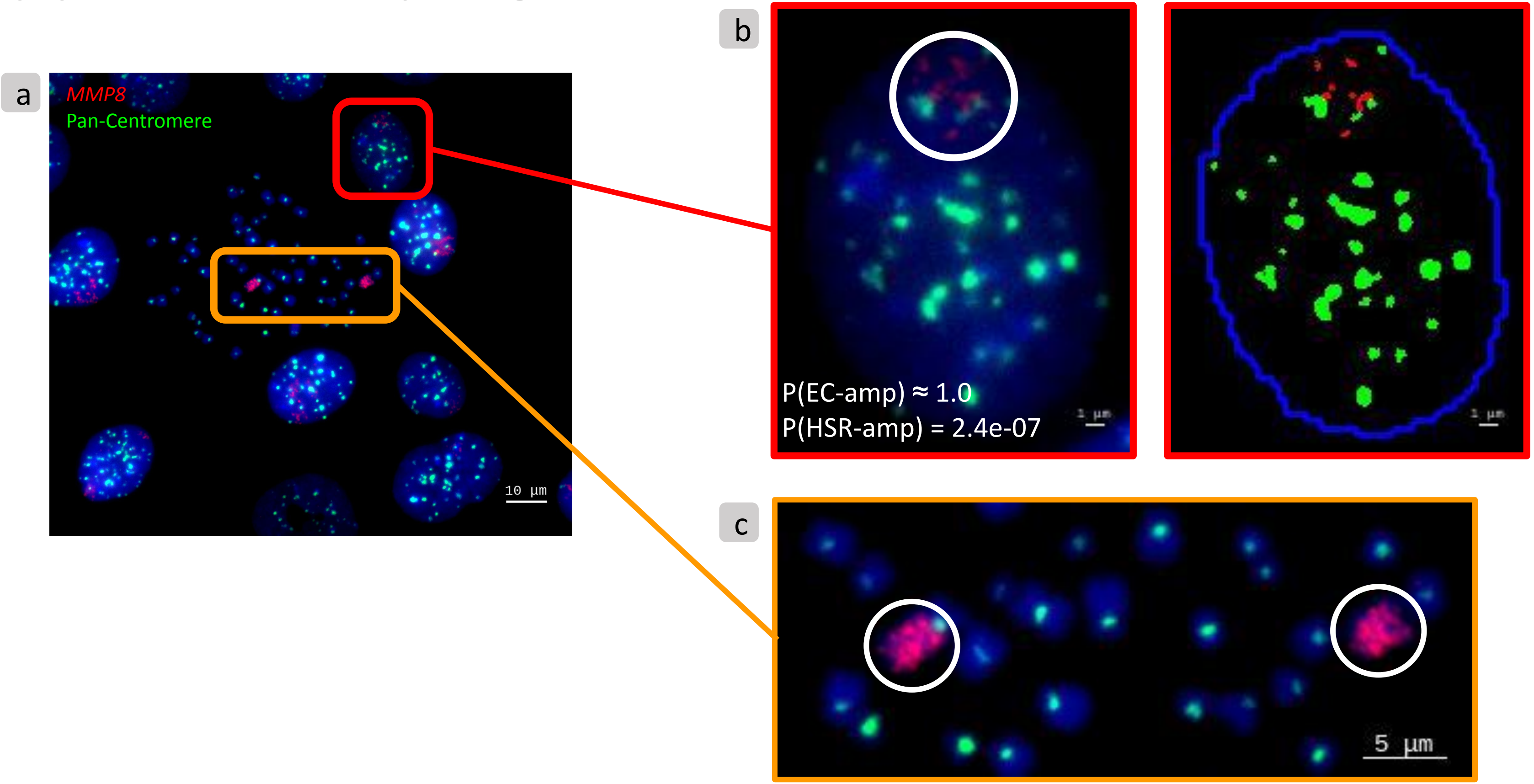

Supplementary Figure 13

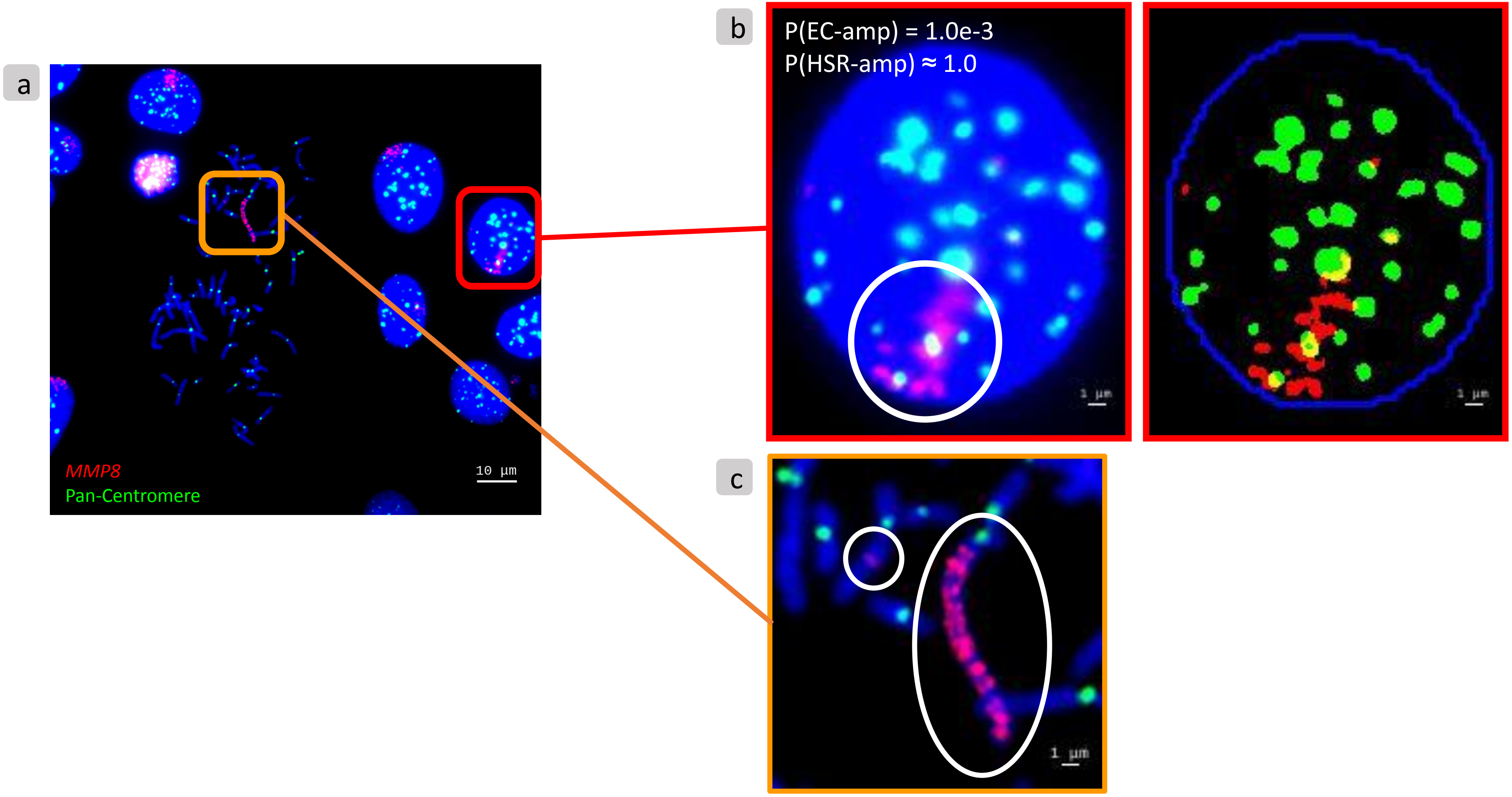

Supplementary Figure 14

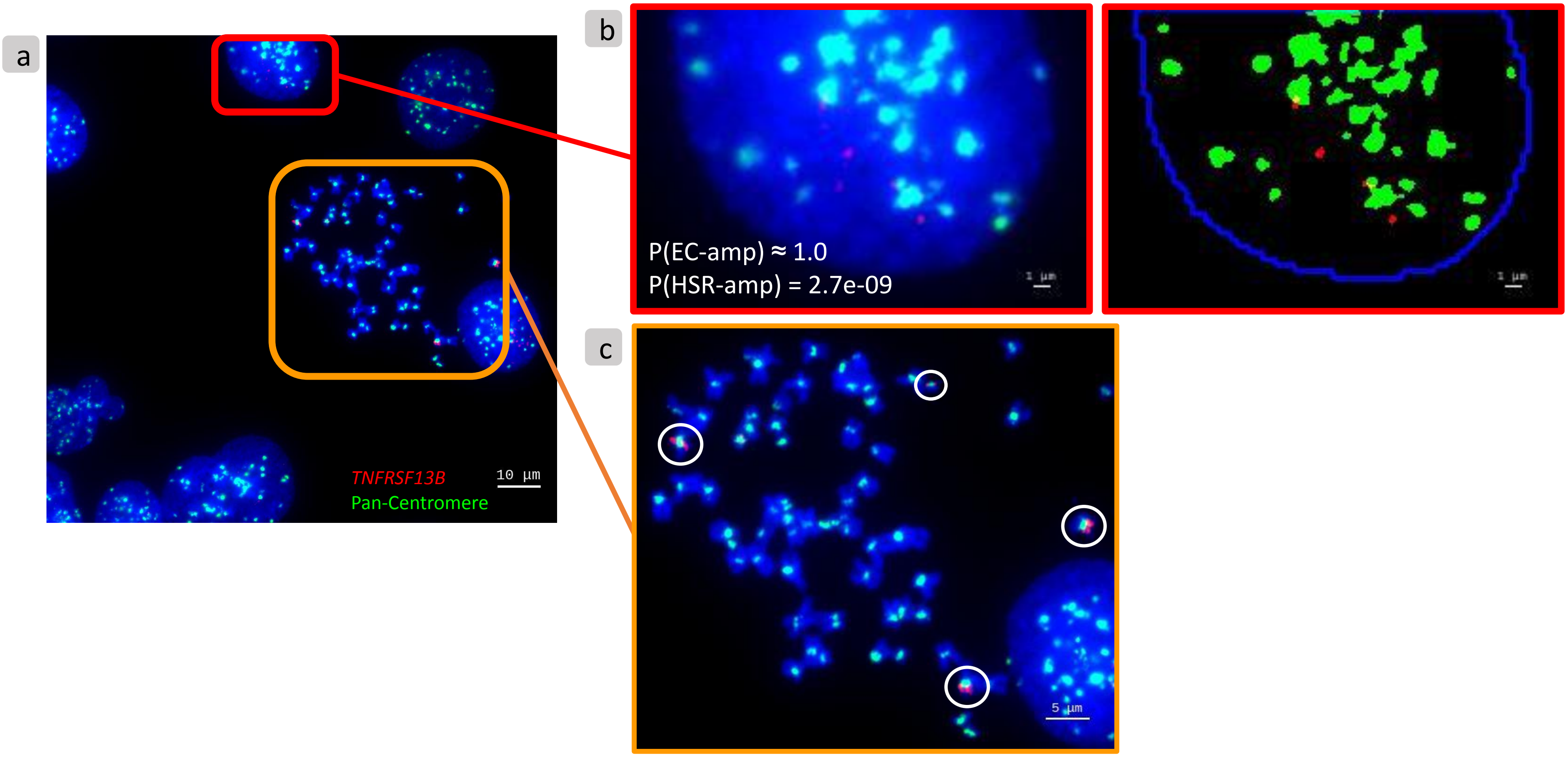

Supplementary Figure 15

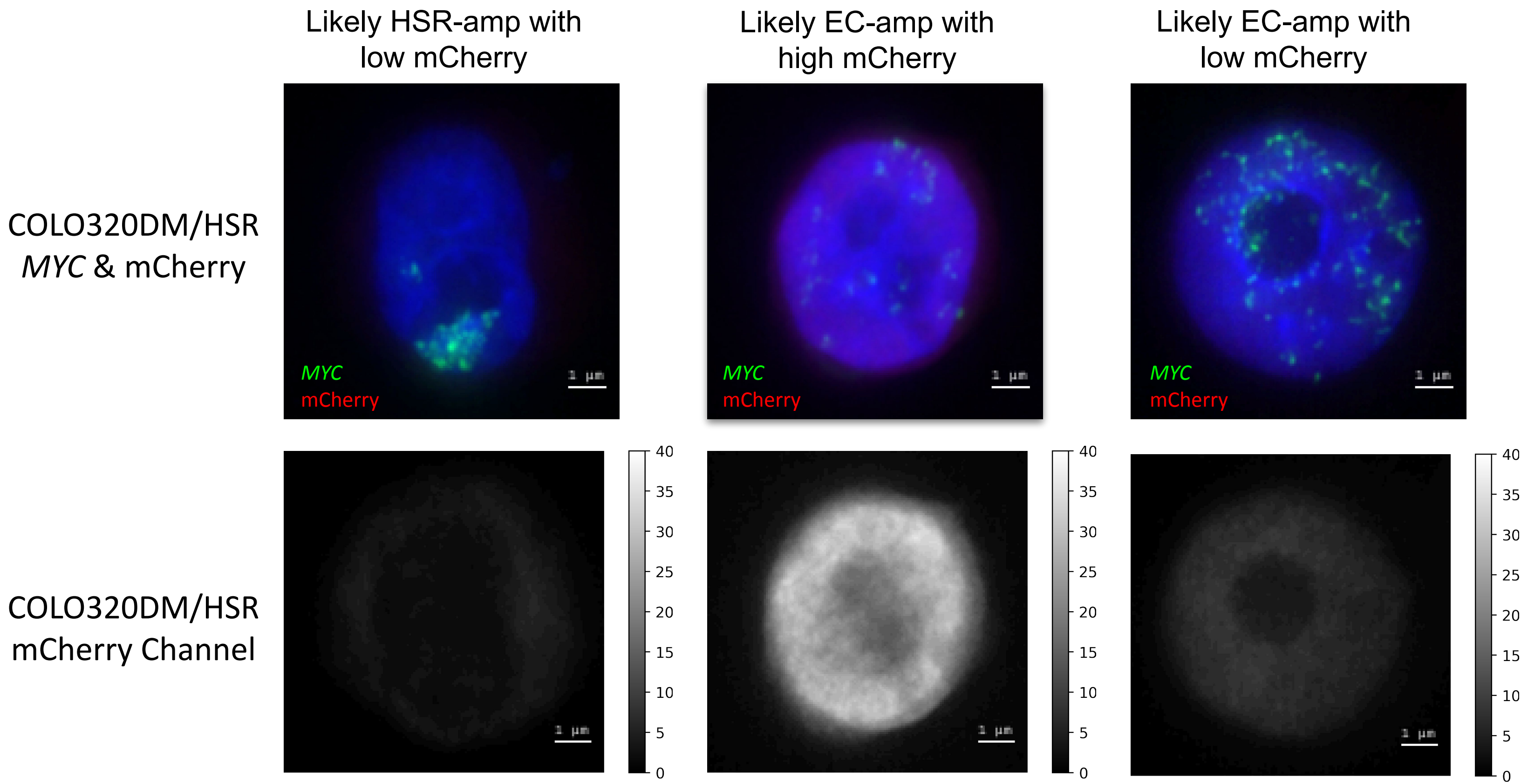

Supplementary Figure 16

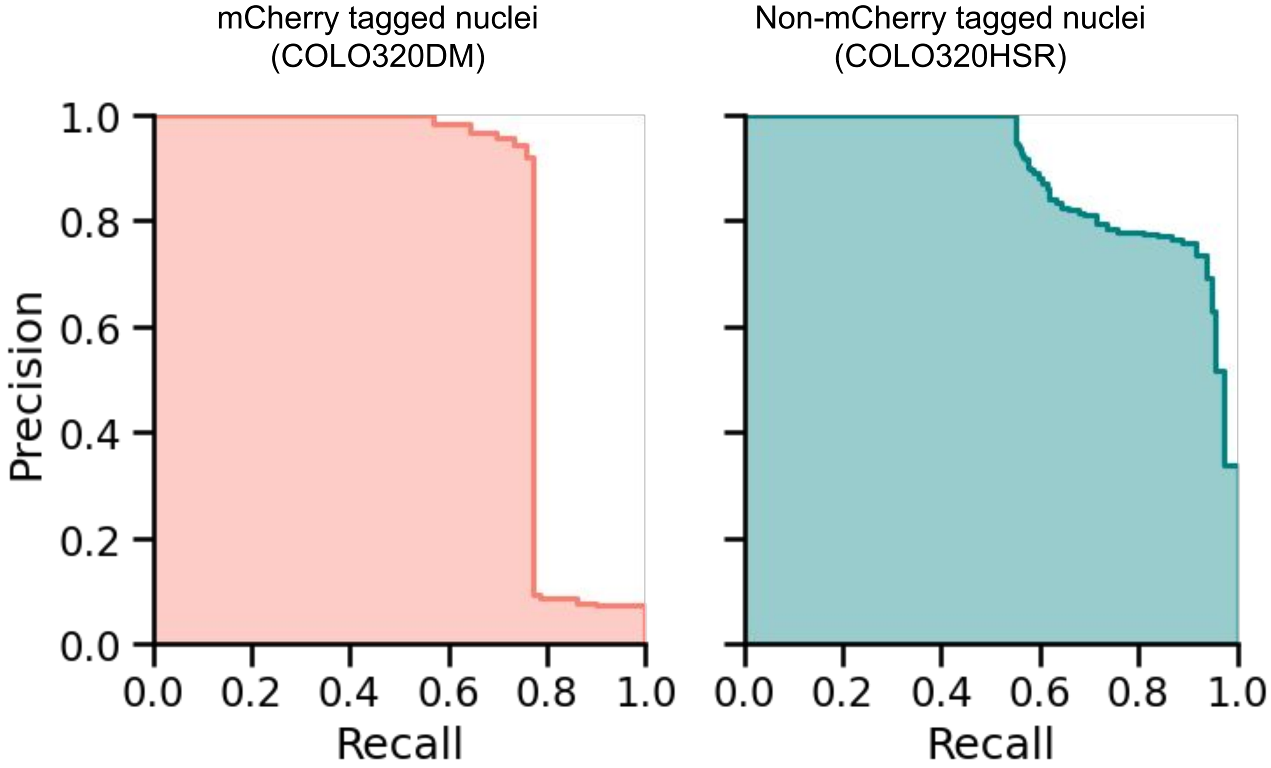

Supplementary Figure 17

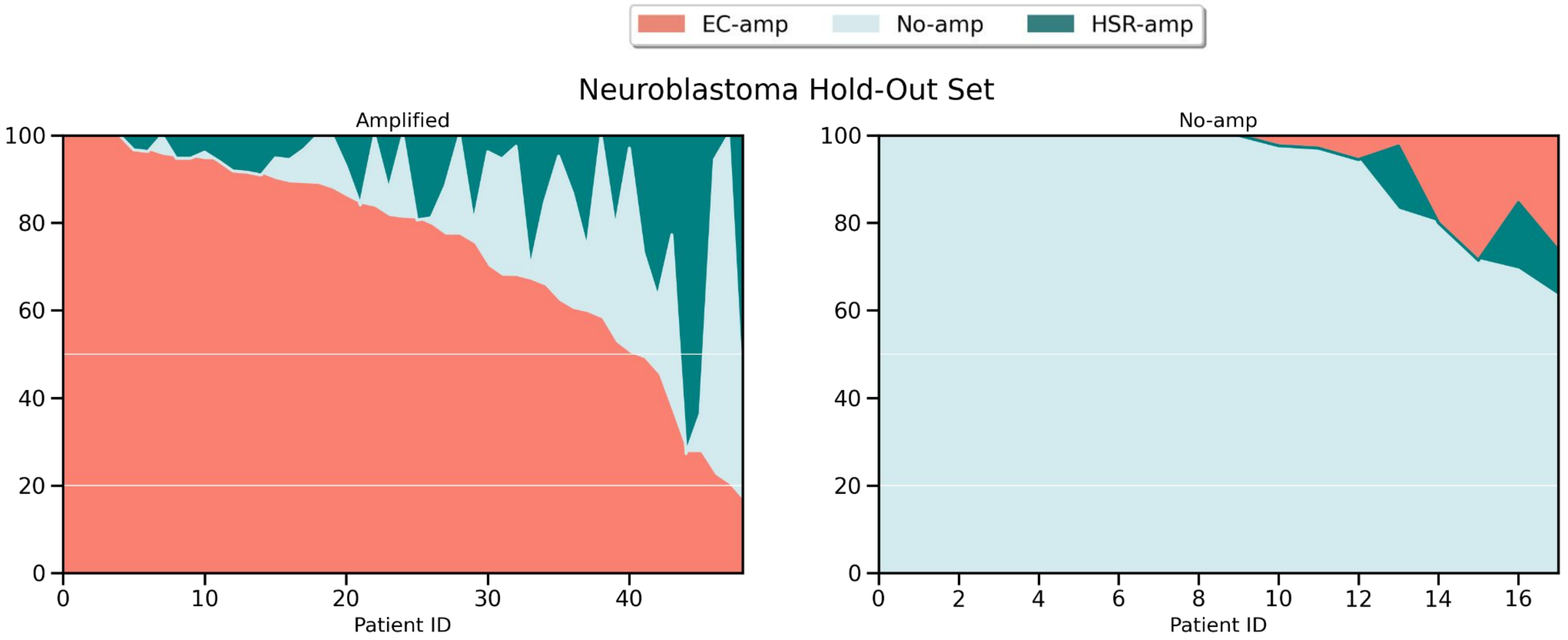

Table of Contents

Supplementary table	Figure reference	Table Caption
ST1	F1	Top table contains the number of images and nuclei from each image acquisition modality and computational mixing experiment. The middle and bottom table contains the breakdown of images and nuclei used for training and testing of ecSeg-i and ecSeg-c respectively.
ST2	F1c	List of cell lines utilized during training and hold-out testing of interSeg. For each cell line, the total count of cultured cell line images and tissue model images is included.
ST3	F4b	Mean and variance of oncogene foci computed for each cell line oncogene pair from the test set, based on stat-FISH results.
ST4	F4e,f	Stat-FISH results for H716 cultured cell images.
ST5	F5b	AmpliconSuite results and interSeg evaluation on neuroblastoma patient tissue image set with WGS data.
ST6	F2b	Grid search results for ecSeg-i, where each row displays a hyperparameter configuration with its corresponding median validation loss. The chosen ecSeg-i hyperparameter choice is bolded. For a single trial, the minimum validation loss was taken across all epochs prior to early stopping. The hyperparameter options include batch size, hidden size, and learning rate. The hidden size variable corresponds to the feature dimension before the final fully connected layer.
ST7	SF2	Grid search results for ecSeg-c, where each row displays a hyperparameter configuration with its corresponding median validation loss. The chosen ecSeg-c hyperparameter choice is bolded. For a single trial, the minimum validation loss was taken across all epochs prior to early stopping. The hyperparameter options include batch size, hidden size, and learning rate. The hidden size variable corresponds to the feature dimension before the final fully connected layer.

ST1. Data Overview

Total	Cultured cells	Tissue models	Patient tissue	"Mixed"	Total
# of images	231	443	265	60	999
# of nuclei	1,903	42,926	7,466	765	53,060

ecSeg-i	Training	Test	Total
# of images	459	215	674
# of nuclei	35,096	9,733	44,829

ecSeg-c	Training	Test	Total
# of images	490	162	652
# of nuclei	28,320	9,438	9,438

ST2. List of Cell Lines Utilized

Cell Line	Tissue	All Oncogenes Probed	Number of Cultured Images	Number of Tissue Model Images
CCFSTTG1	Brain	CDK6,MDM2	0	49
CHP212	Brain	MYCN	0	22
COLO320DM	Colon	MYC	0	41
COLO320HSR	Colon	MYC	20	44
DLD1	Colon	MYC	0	20
DU145	Prostate	NFKBIA	10	0
EKVX	Lung	RORC	10	0
GBM39DN	Brain	EGFR	10	0
GBM39EC	Brain	EGFR	8	0
GBM39HSR	Brain	EGFR	4	0
GSC11	Brain	EGFR	10	0
H2170	Lung	ERBB2,MYC	0	42
H322	Lung	SBDS	10	0
H522	Lung	TNF	10	0
H716	Intestine	FGFR2,MYC	20	0
HCC1569	Breast	BMP5	10	0
HCC827	Lung	EGFR	0	22
HK359	Brain	ARID5B,EGFR	20	0
HOP62	Lung	ERCC2	9	0
KATOIII	Stomach	FGFR2	0	21
MST0211H	Lung	MYC	10	0
NHDF	Skin	MYC	0	21
OVCAR5	Ovary	PAF1	10	0
PC3	Prostate	MYC	10	0
RPMI8226	Blood	IRF4	10	0
RWPE1	Prostate	MYC	0	23
SF268	Brain	MMP8	10	0
SJSA1	Bone	CDK4,MDM2	0	39
SKBR3	Breast	ERBB2,MYC	0	55
SN12C	Kidney	TNFRSF13B	10	0
SNU16	Stomach	FGFR2,MYC	10	44
SW620	Colon	IGFBP1	10	0

ST3. EC-amp vs HSR-amp Sample Level statFISH

Cell Line	Probe	Amp Status	Prediction with Optimal Cutoffs: (Mean Cutoff=9.06, Var Cutoff=48.34)	Mean	Var	n
CCFSTTG1	CDK6	EC-amp	HSR-amp	6.043419267	45.78887308	737
GBM39DN	EGFR	EC-amp	EC-amp	39.66666667	774.5057471	30
CCFSTTG1	MDM2	EC-amp	EC-amp	9.708571429	50.88628135	525
COLO320DM	MYC	EC-amp	EC-amp	9.3359375	92.77602116	128
SNU16	FGFR2	EC-amp	HSR-amp	6.043478261	54.29297659	391
MST0211H	MYC	EC-amp	EC-amp	25.38392857	1101.121541	112
GBM39EC	EGFR	EC-amp	EC-amp	32.1147541	409.6032787	61
CHP212	MYCN	EC-amp	HSR-amp	6.757841907	39.03048177	797
H2170	ERBB2	EC-amp	EC-amp	9.739229025	116.6113894	441
H2170	MYC	EC-amp	HSR-amp	8.394039735	62.88408396	302
H716	FGFR2	EC-amp	EC-amp	48	897.4	21
H716	MYC	EC-amp	EC-amp	43.33333333	899.1764706	18
PC3	MYC	EC-amp	EC-amp	61.55	1717.484615	40
SNU16	MYC	EC-amp	EC-amp	9.887445887	142.3256989	462
HK359	EGFR	EC-amp	EC-amp	41.94117647	1063.496471	51
H322	SBDS	HSR-amp	HSR-amp	7.95	153.3307692	40
GSC11	EGFR	HSR-amp	HSR-amp	1.727272727	2.329545455	33
GBM39HSR	EGFR	HSR-amp	HSR-amp	13.78947368	28.96842105	76
EKVX	RORC	HSR-amp	HSR-amp	5.041666667	28.12862319	24
KATOIII	FGFR2	HSR-amp	HSR-amp	1.132450331	1.356438948	755
COLO320HSF	MYC	HSR-amp	HSR-amp	2.733920705	17.19368964	1135
SW620	IGFBP1	HSR-amp	HSR-amp	5.056603774	23.05442671	53
H522	TNF	HSR-amp	HSR-amp	4.530612245	80.42091837	49
DU145	NFKBIA	HSR-amp	HSR-amp	3.477272727	62.76691332	44
HCC1569	BMP5	HSR-amp	HSR-amp	3.476190476	7.865272938	42
SF268	MMP8	HSR-amp	HSR-amp	8.264150943	90.73657475	53
HOP62	ERCC2	HSR-amp	EC-amp	19.76923077	3252.025641	13
HCC827	EGFR	HSR-amp	HSR-amp	1.394504416	2.156590594	1019
SJSA1	CDK4	HSR-amp	HSR-amp	3.119485294	8.400061613	544
OVCAR5	PAF1	HSR-amp	HSR-amp	4.341463415	102.9804878	41
RPMI8226	IRF4	HSR-amp	EC-amp	12.52631579	558.075188	57
SN12C	TNFRSF13B	HSR-amp	HSR-amp	2.448979592	15.79421769	49
SKBR3	MYC	HSR-amp	HSR-amp	6.669879518	40.66128863	415
SKBR3	ERBB2	HSR-amp	HSR-amp	8.775743707	63.46335524	437
SJSA1	MDM2	HSR-amp	HSR-amp	4.699040767	12.35709392	834
HK359	ARID5B	HSR-amp	HSR-amp	3.923076923	9.353846154	26
RWPE1	MYC	No-amp	No Prediction	2.853372434	17.40871842	682
NHDF	MYC	No-amp	No Prediction	3.871657754	23.73683531	374
DLD1	MYC	No-amp	No Prediction	1.464776632	1.679755668	1164

ST4. H716

image_name	nucleus_center	#_FISH_pixels (green)	#_FISH_foci (green)	Avg fish intensity (green)	Max fish intensity (green)	#_FISH_pixels (red)	#_FISH_foci (red)	Avg fish intensity (red)	Max fish intensity (red)	#_DAPI_pixels	#_FISH_pixels (green and red)	#_FISH_foci (green and red)
H716_ecDNA_FGFR2_green 5	25_1020	725	11	61.2695621	255	390	10	47.37772194	193	8358	124	5
H716_ecDNA_FGFR2_green 5	219_820	2758	88	73.9312525	255	2326	72	59.155795	211	22459	210	16
H716_ecDNA_FGFR2_green 5	416_518	3618	83	95.34320673	255	3091	49	81.77998328	255	22721	584	33
H716_ecDNA_FGFR2_green 5	406_1102	286	10	43.53072134	223	807	22	42.74410414	226	15901	54	4
H716_ecDNA_FGFR2_green 5	429_723	3425	81	97.65353821	255	2758	57	78.81413431	255	21607	488	29
H716_ecDNA_FGFR2_green 5	698_448	3234	30	112.3215961	255	2237	28	90.90675241	255	18971	668	21
H716_ecDNA_FGFR2_green 5	935_717	249	6	40.48186275	248	227	9	34.84101307	145	6120	0	0
H716_ecDNA_FGFR2_green 2	88_266	3709	91	104.4556281	255	4929	83	101.5623043	255	29388	694	28
H716_ecDNA_FGFR2_green 2	379_907	3409	108	84.91917636	255	3917	65	73.24449922	255	38949	298	17
H716_ecDNA_FGFR2_green 1	171_1362	193	8	50.89662767	255	318	12	39.00137646	191	7265	0	0
H716_ecDNA_FGFR2_green 1	403_1217	3010	57	109.853916	255	3041	45	91.2608579	255	22357	403	19
H716_ecDNA_FGFR2_green 1	511_754	3226	79	74.13567027	255	1775	60	33.75146528	252	26616	202	15
H716_ecDNA_FGFR2_green 1	731_1010	1822	33	91.60065565	255	1984	38	62.33705733	255	23183	238	13
H716_ecDNA_FGFR2_green 1	844_298	970	32	48.98419326	255	2938	57	55.35043245	255	26824	80	6
H716_ecDNA_FGFR2_green 6	40_746	3876	49	134.3651661	255	3624	37	135.2435889	255	16105	1306	35
H716_ecDNA_FGFR2_green 6	43_990	1912	45	69.34950341	255	1575	27	61.4928721	255	14801	212	17
H716_ecDNA_FGFR2_green 6	649_580	1363	28	54.66204526	255	1001	29	47.26070039	255	26471	40	3
H716_ecDNA_FGFR2_green 8	61_36	820	19	56.70464403	209	1100	35	53.30413625	188	9453	91	8
H716_ecDNA_FGFR2_green 8	241_1153	2315	34	141.4479015	255	2777	27	131.2079098	255	14463	392	22
H716_ecDNA_FGFR2_green 8	277_73	1852	61	47.95044578	255	3877	87	64.46613449	255	28938	342	23
H716_ecDNA_FGFR2_green 8	352_724	1738	43	59.20691785	255	2611	47	64.79198936	255	21047	332	17
H716_ecDNA_FGFR2_green 8	516_1312	230	8	39.52603679	255	0	0	20.69608608	46	6414	0	0
H716_ecDNA_FGFR2_green 9	454_950	5374	119	102.0216907	255	1838	38	50.05066342	255	38588	128	10
H716_ecDNA_FGFR2_green 9	431_573	468	24	66.68966684	229	213	7	55.81227803	255	5913	36	2
H716_ecDNA_FGFR2_green 9	440_766	752	29	70.44480969	239	178	9	49.87612457	255	5780	58	5
H716_ecDNA_FGFR2_green 9	904_954	1844	40	63.16672565	255	3334	60	67.09362633	255	31081	102	7
H716_ecDNA_FGFR2_green 9	934_421	1453	40	74.8239041	255	1612	34	66.30728755	255	25276	89	5
H716_ecDNA_FGFR2_green 3	205_41	409	15	37.67695826	167	840	23	39.12642939	230	13992	34	3
H716_ecDNA_FGFR2_green 3	548_689	1723	55	47.36321539	255	784	31	29.98341051	192	22243	49	3
H716_ecDNA_FGFR2_green 3	613_47	2080	47	60.97722112	255	533	24	33.09388305	161	14092	119	7
H716_ecDNA_FGFR2_green 3	832_1103	2293	32	122.1170775	255	1437	19	94.30570624	255	14563	224	13
H716_ecDNA_FGFR2_green 3	886_948	1630	39	144.4283505	255	1590	39	144.2088982	255	10991	277	19
H716_ecDNA_FGFR2_green 4	255_235	2002	60	60.80483255	255	768	18	52.21625022	255	23052	26	3
H716_ecDNA_FGFR2_green 4	516_610	1009	25	69.40051866	255	1518	31	83.5790232	255	13882	138	7
H716_ecDNA_FGFR2_green 4	554_272	3365	54	113.9494051	255	2085	37	95.96019126	255	17986	437	21
H716_ecDNA_FGFR2_green 7	471_1090	4386	121	112.9137412	255	2707	44	65.66857252	255	26235	560	28
H716_ecDNA_FGFR2_green 7	478_812	2296	91	92.16019881	255	4695	83	93.6728827	255	25150	319	19
H716_ecDNA_FGFR2_green 7	832_863	1677	72	97.04388478	255	3495	52	99.27250792	255	19893	292	18
H716_ecDNA_FGFR2_green 7	934_1046	1014	32	69.14791197	255	2470	42	56.2354771	255	25718	80	6
H716_ecDNA_FGFR2_green 7	971_66	919	24	52.69427083	255	257	11	23.13637153	198	11520	22	2
H716_ecDNA_FGFR2_green 10	167_429	231	8	47.99784814	177	116	6	29.88395327	140	6506	0	0
H716_ecDNA_FGFR2_green 10	617_1039	2756	100	59.75606459	255	3422	117	40.64243233	255	55486	109	9
H716_ecDNA_FGFR2_green 10	816_1327	2234	41	125.2543978	255	2870	43	117.2883562	255	15519	473	14

ST5. NB Patient Tissue interSeg and AS results

sample_name	AS *MYCN* Prediction	interSeg HSR-amp (%)	interSeg EC-amp (%)	interSeg No-amp (%)	n	test_status	tgt_qscore	cen_qscore	Associated NB Sample Name	Pathologist_Annotation
NBL31_MYCN_CEP2	No-amp	0	0	100	7	TRUE	0.8095403079	-0.7617520525	N/A	N/A
CB2022_NMYC_CEP2	ecDNA	0	80.76923077	19.23076923	26	TRUE	0.5488206154	0.5445104547	BD	Amplification
NBL34_MYCN_CEP2	ecDNA	30.55555556	66.66666667	2.777777778	36	TRUE	0.4716613157	-0.7005118879	MT	Amplification
CB2001_MYCN_CEP2	ecDNA	3.846153846	94.23076923	1.923076923	52	TRUE	0.2947358622	-0.4459107604	MD	Amplification
CB2027_MYCN_CEP2	ecDNA	7.352941176	88.23529412	4.411764706	68	TRUE	0.7567123328	2.396000376	N/A	N/A
CB2034_MYCN_CEP2	ecDNA	5.263157895	94.73684211	0	19	TRUE	0.5834883366	0.07987635129	IK	Amplification
NBL26_MYCN_CEP2	ecDNA	22.22222222	62.96296296	14.81481481	27	TRUE	0.5582687508	-0.5405444427	MC	Amplification
CB2025_MYCN_CEP2	ecDNA	48.88888889	42.22222222	8.888888889	45	TRUE	0.8465002266	0.464256881	L	Amplification
NBL42_MYCN_CEP2	ecDNA	4.545454545	63.63636364	31.81818182	66	TRUE	0.5372883633	-0.3483050244	N/A	N/A
NBL29_MYCN_CEP2	ecDNA	4.545454545	81.81818182	13.63636364	44	TRUE	0.8381059177	-0.7207792052	N/A	N/A
CB2013_MYCN_CEP2	ecDNA	6.451612903	93.5483871	0	31	TRUE	0.7493063798	3.332790653	N/A	N/A

ST6. ecSeg-i Grid Search

Batch Size	Learning Rate	Hidden Dimension	Validation Loss Trial 1	Validation Loss Trial 2	Validation Loss Trial 3	Median Validation Loss (n=3)
**32**	**0.0005**	**128**	**0.190983057**	**0.186280489**	**0.1813333482**	**0.186280489**
32	0.001	128	0.1877988726	0.1960916966	0.1841081977	0.1877988726
32	0.0005	64	0.1898013651	0.1857826263	0.2245612293	0.1898013651
32	0.001	64	0.1820590347	0.190266192	0.2040677369	0.190266192
32	0.0005	32	0.2180010229	0.1932177693	0.1814241409	0.1932177693
64	0.001	64	0.2026931047	0.1828574538	0.1955162138	0.1955162138
32	0.001	32	0.1957267225	0.203797698	0.1934096962	0.1957267225
32	0.005	32	0.2890124321	0.1945998967	0.1974684298	0.1974684298
16	0.005	64	0.2447267473	0.198307395	0.1928061247	0.198307395
64	0.0005	128	0.2122683674	0.2005033791	0.1985675544	0.2005033791
16	0.0005	128	0.1990655065	0.2200064957	0.2025770843	0.2025770843
16	0.001	32	0.2293822169	0.1880499721	0.2041413784	0.2041413784
16	0.0005	32	0.2430012077	0.206753999	0.1851963997	0.206753999
64	0.001	128	0.2096541077	0.2097177356	0.1917420626	0.2096541077
64	0.001	32	0.2100289315	0.1996363252	0.2175378501	0.2100289315
64	0.0005	32	0.2107326239	0.2293731272	0.1979650706	0.2107326239
16	0.005	128	0.217385903	0.2190455049	0.2514328957	0.2190455049
16	0.001	128	0.2270982862	0.2144049555	0.2205274105	0.2205274105
64	0.0005	64	0.2257290781	0.2208190113	0.1875579953	0.2208190113
16	0.005	32	0.1981303096	0.2295539975	0.2255814523	0.2255814523
16	0.001	64	0.2262188494	0.2161845565	0.2284111083	0.2262188494
16	0.0005	64	0.2226154953	0.2275923193	0.2352297604	0.2275923193
64	0.005	128	0.2735762894	0.2307618856	0.2370642126	0.2370642126
32	0.005	64	0.2206793576	0.4172438979	0.2394623756	0.2394623756
64	0.005	64	0.234945327	0.2500035167	0.2407101244	0.2407101244
64	0.005	32	0.3774266839	0.310687989	0.2236115336	0.310687989
32	0.005	128	0.3901348412	1.257973075	0.1937008202	0.3901348412

ST7. ecSeg-c Grid Search

Batch Size	Learning Rate	Hidden Dimension	Validation Loss Trial 1	Validation Loss Trial 2	Validation Loss Trial 3	Median Validation Loss (n=3)
**64**	**0.0005**	**128**	**0.1430557221**	**0.1751743555**	**0.13728787**	**0.1430557221**
64	0.0005	64	0.1436352879	0.1718527973	0.145147711	0.145147711
64	0.001	128	0.1537866294	0.1937006563	0.1493778378	0.1537866294
32	0.0005	64	0.1541920006	0.1456936747	0.1810413003	0.1541920006
32	0.0005	32	0.1606687009	0.1624099463	0.1665202379	0.1624099463
16	0.0005	32	0.1577410251	0.1760385334	0.1629436761	0.1629436761
32	0.0005	128	0.1453581303	0.1649539173	0.1978397369	0.1649539173
32	0.001	128	0.1722031683	0.2011141479	0.1642578542	0.1722031683
64	0.001	64	0.16395621	0.2231697291	0.1752681434	0.1752681434
64	0.0005	32	0.2101816684	0.144564718	0.1888438463	0.1888438463
16	0.0005	128	0.1878876239	0.2311222255	0.1890403777	0.1890403777
16	0.0005	64	0.1893880665	0.182908535	0.1923092902	0.1893880665
16	0.001	32	0.2268929631	0.1951076537	0.2092869729	0.2092869729
16	0.001	64	0.2120130658	0.2261529863	0.1918755919	0.2120130658
64	0.001	32	0.2137893736	0.1764202565	0.2361067533	0.2137893736
16	0.001	128	0.2181571424	0.1530054063	0.2254360467	0.2181571424
32	0.001	32	0.2076869905	0.2296333015	0.2219842225	0.2219842225
32	0.001	64	0.2338222414	0.235560447	0.1720037162	0.2338222414
32	0.005	64	0.234686777	0.2253950834	0.2555097938	0.234686777
16	0.005	64	0.3078441024	0.2328256369	0.238093555	0.238093555
64	0.005	128	0.2595003247	0.2459205836	0.2523165941	0.2523165941
16	0.005	128	0.2604321837	0.2570520937	0.2739439905	0.2604321837
32	0.005	128	0.4244009554	0.2402547747	0.2640328407	0.2640328407
64	0.005	32	0.2699286342	0.2657060325	0.2483081073	0.2657060325
32	0.005	32	0.2437672317	0.6929662228	0.2783691585	0.2783691585
64	0.005	64	0.3186497092	0.2302756011	0.308467418	0.308467418
16	0.005	32	0.2386582643	0.6914008856	0.6920062304	0.6914008856
