# Supplementary material for: Accurate prediction of ecDNA in interphase cancer cells using deep neural networks
Source: Commun Biol. 2026 Apr 11;9:805. doi: 10.1038/s42003-026-09982-4 (PMC13265960; doi:10.1038/s42003-026-09982-4)
Supplement: Supplementary file 2 — Description of Additional Supplementary Files [file 42003_2026_9982_MOESM2_ESM.docx]

**Description of Additional Supplementary File**
File name: Supplementary Data 1
Description: InterSeg evaluation on neuroblastoma patient tissue images.

File name: Supplementary Data 2
Description: InterSeg evaluation on the cultured cell line and tissue model test set.

File name: Supplementary Data 3
Description: Mean and variance computed for every cultured cell and tissue model image, based on stat-FISH results.

File name: Supplementary Data 4
Description: Stat-FISH results for cultured cell line and tissue model experiments with EC-amp.

File name: Supplementary Data 5
Description: Stat-FISH results for cultured cell line and tissue model experiments with HSR-amp.

File name: Supplementary Data 6
Description: Stat-FISH results for cultured cell line and tissue model experiments with no-amp.

File name: Supplementary Data 7
Description: Precision and recall scores on the hybrid COLO320DM and COLO320HSR plate. For each choice of mCherry max brightness threshold, nuclei that were mCherry tagged and not mCherry tagged were treated as true EC-amplified and true HSR-amplified nuclei, respectively.
