# Supplementary material for: Accurate prediction of ecDNA in interphase cancer cells using deep neural networks
Source: Commun Biol. 2026 Apr 11;9:805. doi: 10.1038/s42003-026-09982-4 (PMC13265960; doi:10.1038/s42003-026-09982-4)
Supplement: Supplementary file 10 — Reporting Summary [file 42003_2026_9982_MOESM10_ESM.pdf]

Corresponding author(s): Vineet Bafna

Last updated by author(s): Mar 5, 2026

Reporting Summary

Nature Portfolio wishes to improve the reproducibility of the work that we publish. This form provides structure for consistency and transparency in reporting. For further information on Nature Portfolio policies, see our Editorial Policies and the Editorial Policy Checklist.

Statistics

For all statistical analyses, confirm that the following items are present in the figure legend, table legend, main text, or Methods section.

n/a	Confirmed	
☐	☒	The exact sample size (*n*) for each experimental group/condition, given as a discrete number and unit of measurement
☐	☒	A statement on whether measurements were taken from distinct samples or whether the same sample was measured repeatedly
☐	☒	The statistical test(s) used AND whether they are one- or two-sided<br>*Only common tests should be described solely by name; describe more complex techniques in the Methods section.*
☐	☒	A description of all covariates tested
☐	☒	A description of any assumptions or corrections, such as tests of normality and adjustment for multiple comparisons
☐	☒	A full description of the statistical parameters including central tendency (e.g. means) or other basic estimates (e.g. regression coefficient) AND variation (e.g. standard deviation) or associated estimates of uncertainty (e.g. confidence intervals)
☐	☒	For null hypothesis testing, the test statistic (e.g. *F*, *t*, *r*) with confidence intervals, effect sizes, degrees of freedom and *P* value noted<br>*Give P values as exact values whenever suitable.*
☒	☐	For Bayesian analysis, information on the choice of priors and Markov chain Monte Carlo settings
☒	☐	For hierarchical and complex designs, identification of the appropriate level for tests and full reporting of outcomes
☐	☒	Estimates of effect sizes (e.g. Cohen's *d*, Pearson's *r*), indicating how they were calculated

Our web collection on statistics for biologists contains articles on many of the points above.

Software and code

Policy information about availability of computer code

Data collection	N/A
Data analysis	interSeg Software (includes ecSeg-c, ecSeg-i, and stat-FISH): https://github.com/UCRajkumar/ecSeg<br>- Version v1.0.0<br>AmpliconArchitect (https://github.com/jluebeck/AmpliconArchitect)<br>- Version 1.4.r2<br>AmpliconClassifier (https://github.com/jluebeck/AmpliconClassifier)<br>- Version 1.2.1<br>AmpliconSuite-pipeline (https://github.com/AmpliconSuite/AmpliconSuite-pipeline)<br>- Version 1.3.4<br>NuSeT (https://github.com/yanglf1121/NuSeT)

For manuscripts utilizing custom algorithms or software that are central to the research but not yet described in published literature, software must be made available to editors and reviewers. We strongly encourage code deposition in a community repository (e.g. GitHub). See the Nature Portfolio guidelines for submitting code & software for further information.

Data

Policy information about availability of data

All manuscripts must include a data availability statement. This statement should provide the following information, where applicable:

- Accession codes, unique identifiers, or web links for publicly available datasets
- A description of any restrictions on data availability
- For clinical datasets or third party data, please ensure that the statement adheres to our policy

All cultured cell line, tissue model, and patient tissue imaging data from our analysis have been made publicly available on Mendeley Data: doi.org/10.17632/t9vmmjg3zc.1. This data repository contains metadata files on train-test split information and model weights. We have uploaded all available images and their associated quality score metrics, including images that do not meet our quality score criterion.

Research involving human participants, their data, or biological material

Policy information about studies with human participants or human data. See also policy information about sex, gender (identity/presentation), and sexual orientation and race, ethnicity and racism.

Reporting on sex and gender	Research findings do not apply to one sex only. Sex was not taken into consideration and was not reported for the majority of the patient images analyzed in this study.
Reporting on race, ethnicity, or other socially relevant groupings	Data on race and ethnicity were not collected.
Population characteristics	This study comprised the analyses of tumour and blood samples of patients diagnosed between 1991 and 2022.
Recruitment	Patients were registered and treated according to the trial protocols of the German Society of Pediatric Oncology and Hematology (GPOH).
Ethics oversight	The collection and use of patient specimens was approved by the institutional review board of the Medical Faculty, University of Cologne. Specimens and clinical data were archived and made available by the National Neuroblastoma Biobank and Neuroblastoma Trial Registry (University Children's Hospital Cologne).

Note that full information on the approval of the study protocol must also be provided in the manuscript.

Field-specific reporting

Please select the one below that is the best fit for your research. If you are not sure, read the appropriate sections before making your selection.

☒ Life sciences ☐ Behavioural & social sciences ☐ Ecological, evolutionary & environmental sciences

For a reference copy of the document with all sections, see nature.com/documents/nr-reporting-summary-flat.pdf

Life sciences study design

All studies must disclose on these points even when the disclosure is negative.

Sample size	We trained and validated interSeg on 652 images (40,446 nuclei). We tested interSeg on 215 cultured cell line and tissue model images (9,733 nuclei), and on 67 neuroblastoma patient tissue samples (1,937 nuclei) for our hold-out set.
Data exclusions	For each image, we generate an oncogenic probe quality score which indicates whether the image is apt for interSeg. We first bin the oncogenic FISH signal into 50 buckets based on their pixel intensities. We then find the highest peak left of the 25th bin and right of the 25th bin. We find the peaks by simply comparing the neighboring values. We compute the quality score, Q, by dividing the leftmost peak (h1) by the rightmost peak (h2), Q=h1/h2. Images with Q <0.2 were marked as low quality.<br><br>Additionally, we excluded nuclei with a mean oncogenic FISH signal below 0.05 from both the interSeg and ecSeg-c analyses, as these nuclei exhibited extremely low oncogenic FISH signal. We generated a centromeric probe quality score for each image as well, based on the kurtosis of the mean centromeric intensity per nucleus. Images with a kurtosis value greater than 3 were marked as having low centromeric probe quality and were excluded from ecSeg-c analysis, defaulting to evaluation in interSeg target-channel-only mode. Additionally nuclei with maximum centromeric pixel intensity less than 10 were also excluded from ecSeg-c analysis and defaulted to the ecSeg-i prediction.
Replication	N/A
Randomization	N/A
Blinding	N/A

Reporting for specific materials, systems and methods

We require information from authors about some types of materials, experimental systems and methods used in many studies. Here, indicate whether each material, system or method listed is relevant to your study. If you are not sure if a list item applies to your research, read the appropriate section before selecting a response.

Materials & experimental systems

n/a	Involved in the study
☒	☐ Antibodies
☐	☒ Eukaryotic cell lines
☒	☐ Palaeontology and archaeology
☐	☒ Animals and other organisms
☒	☐ Clinical data
☒	☐ Dual use research of concern
☒	☐ Plants

Methods

n/a	Involved in the study
☒	☐ ChIP-seq
☒	☐ Flow cytometry
☒	☐ MRI-based neuroimaging

Eukaryotic cell lines

Policy information about cell lines and Sex and Gender in Research

Cell line source(s)	The following cell lines were purchased from ATCC: RWPE1, H716, MSTO211H, SNU16, CCFSTTG1, H2170, COLO320DM, COLO320HSR, H522, HCC1569, KATOIII, SKBR3, SJSA-1, DU145, SW620, SN12C, SF-268. Normal human dermal fibroblast (NHDF) was purchased from Lonza. GBM39DN, GBM39EC and GBM39HSR neurosphere culture were derived from a patient with glioblastoma undergoing surgery at Mayo Clinic, Rochester, Minnesota as described previously (PMID: 16609043). HK359 neurosphere culture was derived from a patient with glioblastoma as described previously (PMID: 27116978). DLD1, HOP62, EKVX, RPMI8226, H522 and OVCAR-5 were part of the NCI-60 cell line panel (gift from A. Shiau, obtained from NCI). H322 was purchased from Sigma. HCC827, CHP212 were from DSMZ-Leibniz Institute. GSC11 was a gift from Dr. Frank Furnari.
Authentication	Cell lines obtained from vendors were not authenticated.
Mycoplasma contamination	Cell lines were tested negative for mycoplasma.
Commonly misidentified lines (See ICLAC register)	None of the cell lines used are registered by ICLAC as commonly misidentified.

Animals and other research organisms

Policy information about studies involving animals; ARRIVE guidelines recommended for reporting animal research, and Sex and Gender in Research

Laboratory animals	Foxn1nu Mice from Charles Rivers at the age of 6 weeks old were used in the study.
Wild animals	This study did not involve wild animals.
Reporting on sex	All Animals were Female
Field-collected samples	The study did not involve samples collected from the field.
Ethics oversight	The animal experiment protocol was approved by and performed in full accordance with the Institutional Animal Care and Use Committee at Stanford University (IACUC protocol number: 34041)

Note that full information on the approval of the study protocol must also be provided in the manuscript.

Plants

Seed stocks	*Report on the source of all seed stocks or other plant material used. If applicable, state the seed stock centre and catalogue number. If plant specimens were collected from the field, describe the collection location, date and sampling procedures.*
Novel plant genotypes	*Describe the methods by which all novel plant genotypes were produced. This includes those generated by transgenic approaches, gene editing, chemical/radiation-based mutagenesis and hybridization. For transgenic lines, describe the transformation method, the number of independent lines analyzed and the generation upon which experiments were performed. For gene-edited lines, describe the editor used, the endogenous sequence targeted for editing, the targeting guide RNA sequence (if applicable) and how the editor was applied.*
Authentication	*Describe any authentication procedures for each seed stock used or novel genotype generated. Describe any experiments used to assess the effect of a mutation and, where applicable, how potential secondary effects (e.g. second site T-DNA insertions, mosiacism, off-target gene editing) were examined.*
